# Spatially restricted occurrence and low abundance as key tools for conservation of critically endangered large antelope in West African savannah

**DOI:** 10.1038/s41598-021-98649-7

**Published:** 2021-09-29

**Authors:** Mallé Gueye, Karolína Brandlová, Thomas Rabeil, Maniang Mamadou Diop, Babacar Diop, Pavla Hejcmanová

**Affiliations:** 1 Direction Des Parcs Nationaux du Sénégal, route des Pères maristes, BP 5135, Dakar Hann, Senegal; 2 Department of HydroSciences and Environment, University Iba Der Thiam de Thiès, Thiès, Senegal; 3 Faculty of Tropical AgriSciences, Czech University of Life Sciences Prague, Kamýcká 129, Prague - Suchdol, 16500 Czech Republic; 4 Wild Africa Conservation, 27 rue de D’Esbly, 77240 Cesson, France

**Keywords:** Zoology, Ecology

## Abstract

The effective conservation of mammals on the brink of extinction requires an integrated socio-ecological approach, yet the updated ecological knowledge of species remains fundamental. This study brings spatiotemporal behaviour, population structure, age-specific survival rates, and population size estimate of the Western Derby eland (WDE) in the Niokolo Koba National Park (NKNP), Senegal, investigated during dry seasons 2017 and 2018. WDE was strongly localised in the core area of NKNP (< 5%), active throughout the day with the highest peak in the hottest daytime, with a mean group size 7.6 ± SE 8.9. The adult sex ratio was female-biased and showed low annual adult male survival rates. The population consisted of high proportion of juveniles, whilst adults did not exceed 40%. The estimated population density was 0.138 WDE/km^2^ (± 0.0102) and estimated size 195 WDE in NKNP (CI95 from 54 to 708 individuals). Findings highlighted that the WDE population has potential to expand in the NKNP, due to an underutilized capacity. The age-specific vital rates indicate adult males as the most vulnerable; suggesting either an increase in the large predators’ population, livestock encroachment pressure, and/or poaching. Findings imply that targeted monitoring with science-based interpretation may bring forward strong conservation solutions to the protected area management decision-makers.

## Introduction

The effective conservation of large mammal species on the brink of extinction requires an integrated socio-ecological approach, linking the ecological requirements and behaviour of the animal with the social, political, and cultural environments surrounding the range of the species’ distribution^1^. Knowledge regarding the true distribution, population size, and life-history of the species remains a fundamental steppingstone to designing successful conservation plans, as managers must identify target areas in which the conservation actions should be focused, and apprehend how (and to what extent) different actions will affect the key vital rates of the population. Understanding the threats that different species face is a prerequisite for effective conservation management, and towards finding appropriate solutions for these. Threats may be of ecological or biological origin, human-induced, or of a broader environmental nature, such as climate change. In many cases, the cause of extinction is due to a combination of threats, creating a species-specific context. These specific threats influence vital rates, are responsible for population performance, and change the population’s trajectory^2^, leading mostly to population decline. Consequently, effective management strategies for endangered species recovery need to be population-specific^3^.

The critically endangered Western Derby eland (*Tragelaphus, syn. Taurotragus derbianus ssp. derbianus*, hereafter reported as WDE), i.e. the western subspecies of the Giant eland^4^, is one of the largest antelope in the world, and yet, direct knowledge regarding its population status and dynamics within its last confirmed refuge, the Niokolo Koba National Park (NKNP) in south-east Senegal, remains virtually unknown. Most of the available information refers to the Eastern subspecies (*T. d. gigas*, hereafter reported as EDE).

The current WDE population size estimate is 120–170 individuals, and is based on reports from numerous irregular wildlife surveys within the park^5–7^. The last direct sighting during an aerial survey recorded a herd of 69 individuals, with no clarification of the existing herd structure^7^. Furthermore, the most recent combined aerial and ground wildlife survey took place in February 2018, and the observed numbers did not allow for an abundance estimation for the WDE^8^, due to the lack of direct sightings. According to former wildlife surveys carried out in the 1960’s and 1970’s, the WDE population number has always been estimated as very low, and never exceeded several hundred individuals^9–12^. To compare, populations of EDE at various sites in Cameroon and Central African Republic are more abundant^13,14^, having several thousands of individuals in total^4^. The conservation actions for WDE have thus had a limited knowledge basis for decision-making, and besides the ecological knowledge acquired on the eastern subspecies, they rely mostly on information gained indirectly from faeces in situ, such as their diet composition^15^, or from studying the foraging strategy, social behaviour, and reproduction of the only ex situ population found within two other natural reserves in Senegal^16–21^.

In order to bring to light the first reliable perspective on the population of the WDE in the NKNP, this study utilized a camera trap monitoring scheme launched in 2017, within the framework of a new ecological monitoring program designed specifically for the Park^22^. Firstly, the study aimed to report the trapping rate, occupancy, and diurnal activity pattern of the WDE in the NKNP, to reveal the spatiotemporal pattern of their behaviour. This information will enable targeted active protection measures, such as law enforcement patrols, as well as ecotourism activities, into zones within the park that are most frequented by the WDE at the most appropriate times of the day. Based on findings on Eastern Derby eland, we expected that animals would range over large distances, either due to browsing on a large variety of wood plant species or in search of water, moving even during hot periods of the day^14^.

Secondly, the study aimed to identify the WDE population structure and group size, based on the individual recognition of animals, and to assess the vital rates and demographic parameters of the WDE population, to clarify the age-specific survival rates. Findings from Cameroon and Central African Republic indicate the social structure of Eastern Derby elands to have a fission–fusion dynamics with variable group size ranging from solitary individuals to tens of individuals, and mixed age/sex composition^13,14^. We therefore expected similar social patterns, while we did not make any assumptions on sex ratio as it may be strongly influenced by predators or human activities in diverse shape.

Thirdly, it aimed to estimate the population size and, lastly, to interpret the status of the population, and the life-history of the WDE in the NKNP, with regards to an environmental and human-related context, its conservation, and management implications.

## Materials and methods

### Study area

**Figure 1:**
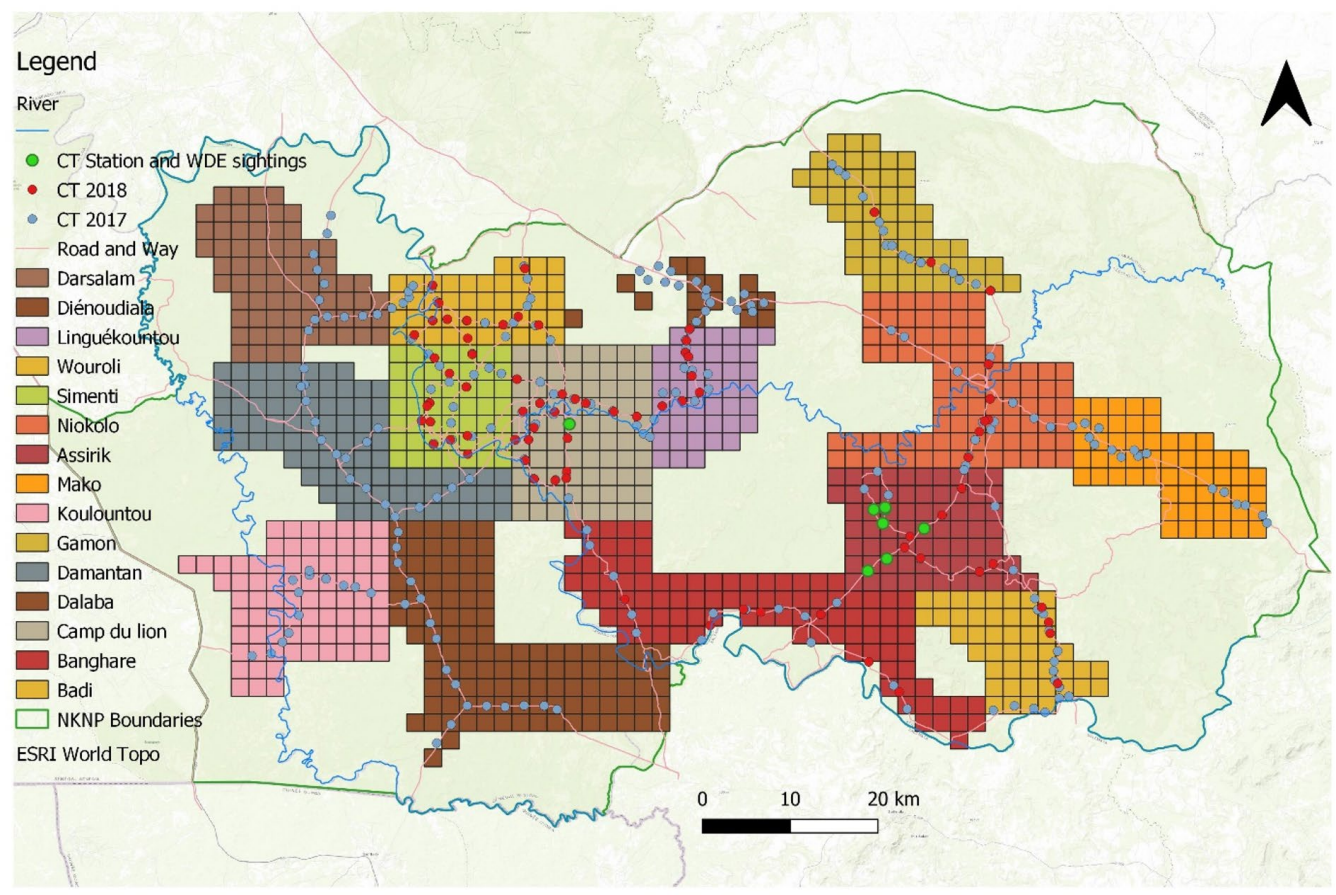
The distribution of the camera traps in the Niokolo Koba National Park in Senegal, in 2018, and the zoning of the park, used to monitor the Western Derby eland population. The map was created with ArcGIS 10.6.1 (https://desktop.arcgis.com/en/).

The NKNP is the largest protected area in Senegal, extending over 9130 km^2^, and located in south-eastern Senegal (Fig. 1). The area was declared a national park in 1954, and was accepted as a Biosphere Reserve, and inscribed on the World Heritage List, in 1981; however, it was only listed as a World Heritage in Danger in 2007^23^.

The Sudan-type climate (with 900–1200 mm of rainfall, a rainy season from June to October, and temperatures ranging from 25 °C in December to 33 °C in May) favours transitional vegetation between the Sudanese and Sudano-Guinean savanna. It corresponds to the tropical climate domain with a dry winter season (Aw), according to the Köppen classification. The vegetation is a mosaic of wooded savanna (dominant tall-stem grass being *Andropogon gayanus*, and tree species being *Piliostigma thonningii*, *Pterocarpus erinaceus*, *Pericopsis africana*, *Bombax costatum*, *Burkea africana*, *Prosopis africana, Strychnos spinosa*), herbaceous savanna (dominant grass: *Cymbopogon giganteus, Schizachyrium sanguineum,* and *Panicum anabaptistum,* and tree species: *Combretum glutinosum*, *C. nigricans*, *C. micranthum*), interspersed by gallery forests (*Borassus aethiopum, Ceiba pentandra*, *Cola cordifolia*, *Detarium senegalense, Khaya senegalensis, Raphia sudanica*) along the Gambia, Koulountou and Niokolo rivers.

There are 80 species of mammals recorded in the NKNP, including nine species of antelope (including roan antelope, *Hippotragus equinus koba;* Western hartebeest *Alcelaphus buselaphus major*; and waterbuck, *Kobus ellipsiprymnus defassa*), West African buffalo (*Syncerus caffer brachyceros*)*,* hippopotamus (*Hippopotamus amphibious*), a few rare individuals of African elephant *(Loxodonta africana)* as the most important large herbivores, West African lions (*Panthera leo leo*), leopards (*P. pardus*), wild dogs (*Lycaon pictus*), spotted hyenas (*Crocuta crocuta*) as the most important carnivores, and baboons (*Papio papio*) as the most abundant primate^7,8^.

### Camera trap settings for detection probability and population size estimates

To calculate the naïve occupancy and trapping rate of WDE in the NKNP, 71 camera traps were placed in the park from January to July 2018, during the dry season, based on the methodology of Rowcliffe et al*.*^24^ and Tobler et al*.*^25^, and the single-season approach^26^. A grid pattern of 4 km^2^ per square was designed over the NKNP area, and camera traps (CTs) were placed on sites in the proximity of gravel roads within the grid (Fig. 1), to assure the long-term regular accessibility. CTs were attached to trees at the height of 50–70 cm, positioned preferentially north or south to avoid directional sunlight, heading towards animal path or waterpoint. Two types of camera traps were used: Dörr (Mini Black 5.0; Neu-Ulm, Germany) and Moultrie (M-888i and L50; Alabama, United States). Photographs were taken after the activation of motion sensors (high sensibility), and every detection resulted in three consecutive pictures, with a minimum interval between events of 15 s and 10 s for the Dörr and Moultrie cameras, respectively. The Dörr CTs and Moultrie M-888i CTs were equipped with an infrared light (low glow), which was automatically activated in poor light conditions, while the Moultrie L50 CTs utilized a white light flash.

Picture Information Extractor (PIE) software (Picmeta Systems 2019; Berlin, Germany) was used to extract metadata information from each photograph of recorded WDE (image name, date, and time). These data were analysed using the Camera Trap Analysis Package (ZSL CTAP; London, England). The “event” was defined as “any sequence for a given species occurring after an interval of ≥ 60 min from the previous three‐image sequence of that particular species”, to ensure that species events were independent^25,27^. The events were automatically screened by the ZSL CTAP software; however, a detailed inspection of all photographs and time periods from the first approaching animal to the last individual of the group was performed. All individuals recorded during one event were considered members of one herd. As the camera traps may not detect all individuals in the herd, due to their limited visual field, the term “group size” used in the results should therefore rather be considered as the “minimum group size”. For calculation of the trapping rate, days when at least 75% of camera are working (from 9th January to 30th June 2018) were used.

To provide a basic evaluation of space-usage in the park, the occupancy and trapping rate for a selected zone within the NKNP was calculated (the zone of Mont Assirik) covering 272 km^2^ (2.97%) of the park (Fig. 1). This zone comprises of two important biotopes; the table mountain of the Mont Assirik with savanna vegetation, and Mansa Fara marsh with permanent water points and lush vegetation of Guinean aspect. There were ten camera traps in this zone, with a total of 1328 operational days. The selection of this zone was based on a pilot CT survey from 2017, and on long-term common observation reports.

### Camera trap settings for other analyses

In addition to the dataset from 2018, 31 CTs were installed during 2016 and 2017, for a period of 30 days in each of the zones of the NKNP^28^ (Fig. 1), during the period between 31st December 2016 and the beginning of July 2017 (Table 1). There were also four additional cameras installed during 2018, outside of the grid deployment. Data from those additional cameras were used for individual identification of the eland, and for the analysis of the social structure of the herds.

**Table 1:** Camera traps which recorded the Western Derby elands in the Niokolo Koba National Park (Senegal), and their operation days during 2016, 2017 and 2018.

Year	Camera trap name	Data collection period	Days deployed	Operational days
2016	Cam 10	04/02 to 12/03/2016	37	37
2017	Mont 6	14/03 to 24/05/2017	71	39
	Mont 7	14/03 to 24/05/2017	71	70
	Mont 11	14/03 to 24/05/2017	71	70
	Mont 12	14/03 to 24/05/2017	71	70
2018	Mont 1	11/01 to 28/06/2018	168	107
	Mont 3	10/01 to 28/06/2018	169	169
	Mont 4	10/01 to 28/06/2018	169	106
	Mont 4’	21/03 to 28/06/2018	99	56

### Circadian activity analyses

To decipher the WDEs’ circadian activity in the NKNP, all available records of WDEs from the CTs during 2016, 2017, and 2018 were used (total of 49 events), and circular statistics were performed with Oriana v4.02 software (Kovach Computing Services 2020; Pentraeth, United Kingdom). First, the mean vector (µ) and the circular standard deviation (CSD) for circadian activity were calculated. Thereafter, the Rayleigh´s Uniformity Test^29^ was used to determine if the data (µ ± CSD) was uniformly distributed (when the test is significant, the data is clumped around a certain date or time). This test is based on the length and direction of the mean vector, and may not be significant when the species has a bimodal circadian activity. The Rao´s Spacing Test^30^ was also performed for the daily activity data, which is based on the uniformity of the spacing between adjacent points. In summary, if any of these tests is significant, it can be concluded that the circadian activity of the species is not uniform.

### Identification of individual animals, population structure and group size

To determine the population structure and size of herds, a detailed inspection of all images from 2016 to 2018 where WDE were recorded and clearly visible, was performed (total of 429 images from 49 events).

All images within one event sequence at the individual animal level were analysed, to obtain as much complete information about the number of animals in the herd as possible. From here onwards, one event is reported as being one herd. Where possible, the sex and age category of each recorded animal was determined in every image, according to external traits such as body size, and horn size and shape^31–33^. The sex categories were: male (M), female (F), and unspecified (U), and the age categories were: adult (AD) for animals ≥ three years old, and with three spirals of their horns; subadult, distinguishing between one-year-old (1Y) animals with straight horns, bearing one spiral at the base, and two-year-old (2Y) animals with two spirals, and a specific V shape of the horns; and juveniles (JUV), up to one year of age, with short and straight horns.

Animals were identified individually when possible, using specific individual traits and the coat pattern, namely the white stripes unique to each animal^16^. The resulting photographs were assessed for the left (L) and right (R) sides of the animals separately, as in most cases it was not possible to visualize both sides. Thus for further analyses, the right sides were used because they were more frequent in the images, as similarly reported by Gosling^34^. The rate of identification (ID) success, i.e. the number of identified animals within the age-sex category divided by the total number of individuals recorded in the respective category, was calculated for all years and events together. General Linear Model (GLM) were used in the TIBCO® Statistica™ package (StatSoft, Palo Alto, CA, USA) to determine if the rate of ID success was affected by the respective age-sex category (ADM, ADF, 2YM, 2YF, 1YM, 1YF, JUV), time of day (DAYLIGHT, NIGHT), CT identity, and group size (total number animals in the event). Animals which were not assigned to a specific age-sex category were omitted from this analysis.

Despite the different CT efforts in 2017 and 2018, the ratio of age classes was comparable between the two periods, and also with other studies^13,14^, it was therefore assumed that there was an equal trapping probability for all age-sex classes. The total number of individuals of each age-sex category recorded during each camera event was then calculated. Subsequently, the herds were classified into three types: herds with mixed sexes (MIX), male-only herds (UNI-M), and female-only herds (UNI-F). The number of individuals in different herd types was compared, as well as the differences between the group size in 2017 and 2018, and between the seasons (dry, hot dry), using GLM (TIBCO® Statistica™ package, StatSoft, Palo Alto, CA, USA). For those analyses, the single individual recorded in 2016 was omitted.

The birth season was estimated based on juvenile horn size (horns are shorter than ears in individuals < four months old)^32^. Given that eland females have one calf per year (with a gestation time range of 255–275 days for Eastern Giant Eland^14^), and assuming a stationary age distribution and equal probability of sampling for all individuals, the vital rates of the WDE population were calculated^13^, despite of the limitations connected with the lack of long-term data about population dynamics^14^. First, the sex ratio of all detected individuals was calculated, separately for juveniles and adults. By dividing the number of JUV by ADF, the breeding rate was estimated, and the number of JUV and 1Y animals was used to calculate annual mortality rates, firstly for the juveniles:

$$ {\text{M}}_{{{\text{anJUV}}}} = \left( {{\text{N}}_{{{\text{JUV}}}} - {\text{N}}_{{{\text{1Y}}}} } \right)/{\text{N}}_{{{\text{JUV}}}} $$

Then, the 1Y and 2Y animals were polled together to obtain the number of subadult animals (SUB), and the annual adult mortality rates for females and males, respectively, were calculated as follows:

$$ {\text{M}}_{{{\text{anADF}}}} = {\text{N}}_{{{\text{SUBF}}}} \times \left( {{1} - {\text{M}}_{{{\text{anJUV}}}} } \right)/{\text{N}}_{{{\text{ADF}}}} $$

$$ {\text{M}}_{{{\text{anADM}}}} = {\text{N}}_{{{\text{SUBM}}}} \times \left( {{1} - {\text{M}}_{{{\text{anJUV}}}} } \right)/{\text{N}}_{{{\text{ADM}}}} $$

### Population size estimates

To estimate the population size of the WDE, the Spatially Explicit Capture Recapture model (SECR) was applied, an advanced method providing the means for robust density estimation while explicitly accounting for spatially-induced individual heterogeneity in detection probability. The spatial element in density estimations is particularly linked to camera trap location with regards to specific species because the location inherently influences the detection probabilities for the species^36,37^. The analyses were performed in R Studio 1.3.959 (R Core Team 2020), using functions implemented in the ‘secr’ package 4.3.0^38^. Specifically, capture histories of ID animals detected in 2018 were used, and one day was considered as a single capture occasion. Each ID individual was captured only once on each occasion, i.e. the recorded recaptures occurred on different occasions. The camera traps were considered proximity sensors^38^. There were 71 CTs used as detectors, with an average spacing of 2315.128 m. To estimate the animal density, home range centres were assumed to follow a 2-D Poisson distribution, and to be fixed for the duration of trapping. The animal density was estimated as a derived parameter from the top AICc‐ranked models for half-normal, exponential, and hazard rate distribution functions^36^. Population size was then estimated as the volume under the density surface. The ‘mask area’ covered by the CTs was calculated automatically^39^. The ‘secr’ package produced an estimate of the probability of detection at the centre of a home range (parameter g0), and of the function of the scale of animal movement (parameter sigma). A null model was generated, in which both g0 and sigma were constant, because no trapping response in the ID animals was expected as they were all photographed within the daylight period, and all animals were known to occupy a limited area within the park, with no long-distance movements expected. The model further assumed that the population was demographically closed during sampling, individuals were correctly identified, and detections were independent^36,40^.

## Results

### Detection probability estimates

The WDE were recorded on 429 images during 49 events over a three-year deployment, more specifically, in one event in 2016, 16 events in 2017, and 35 events in 2018. During the dry season in 2018, the naïve occupancy of WDE was 0.048, and the trapping rate was 0.348 per 100 CT days in the NKNP. Focusing on the zone of the Mont Assirik and Mansa Fara, the occupancy increased to 0.31, and the trapping rate was 2.41 per 100 CT days.

### Circadian activity pattern

**Figure 2:**
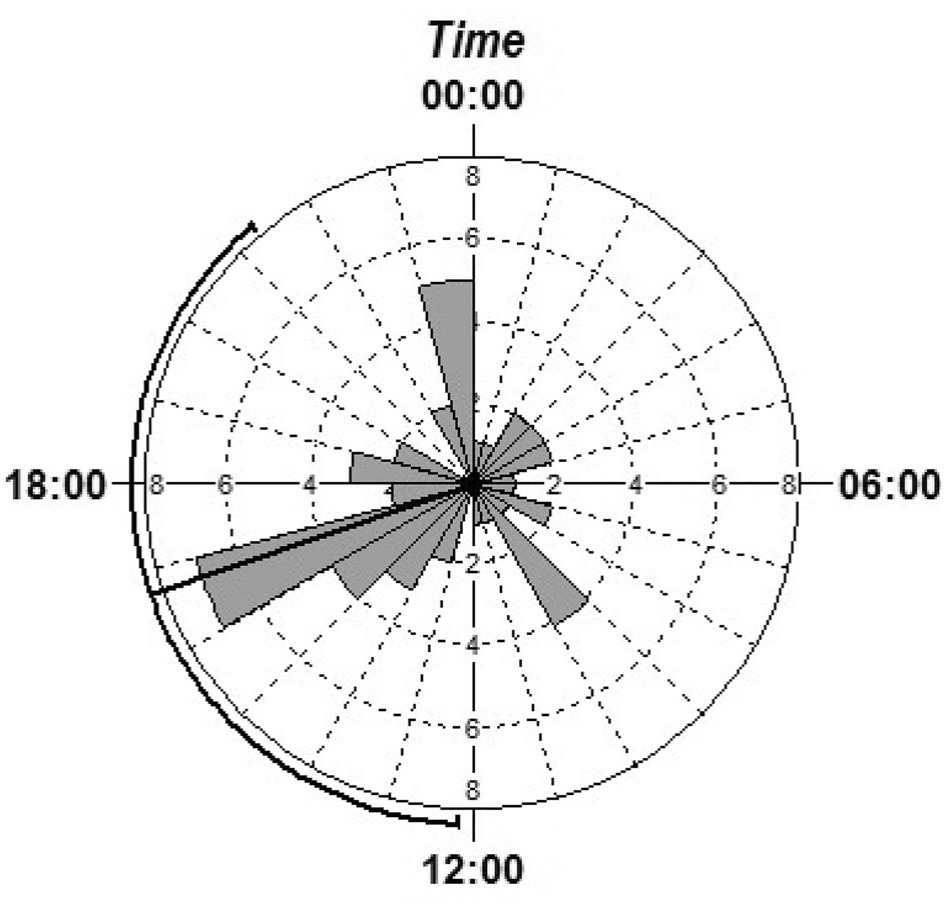
Circadian activity of the Western Derby elands in the Niokolo Koba National Park, Senegal. Each wedged-shaped column of histogram indicates the number of events at the indicated time, while the black line indicates the mean activity vector and 95% confidence limits.

The WDE were recorded throughout the day, showing that their activity was not concentrated into one activity peak during the day (µ = 16:44; circular standard deviation CSD = 07:15, median = 16:27, Rayleigh uniformity test Z = 1.32, *P* = 0.27), as animals were active throughout the day (Rao´s Spacing Test U = 147, 0.10 > *P* > 0.05). High levels of activity appeared in three peaks during the day; the animals were the most active in the afternoon, specifically between 16:00 and 17:00 when high daily temperatures culminate, then just before midnight, and in the morning, between 9:00 and 10:00 (Fig. 2).

### Identification of individuals and recaptures

First, individuals were assigned into respective sex and age categories. This was possible in 67% of cases, ranging from 0 to 100% assigned animals per event. Then individual identification was done, where 23% of individuals per event were identified on average, of which most (95.6%) were also assigned into an age and sex category.

Twenty-nine individuals were identified from the left side, and 36 individuals from the right side. The probability of successful identification was influenced by the age-sex category (F(7, 126) = 4.804, *P* < 0.001) and by the daytime (F(1,126) = 6.476, *P* < 0.01). This means that when an ADM was recorded in the herd, it was possible to identify him in 44.1% of all cases, which is similar to the identification rate for ADF, being 43.7% of all cases. The lowest rate of individual identification was recorded for 1Y and JUV animals. Animals were more successfully identified during the daytime than during the night hours. Neither the total number of animals per event nor the position of the CT had a significant effect on the identification success rate.

In total, recaptures were recorded in 17% and 15% of ID animals from the right and left side, respectively, with the mean time between recaptures being 127 days (ranging from 0 to 787 days). Recaptures were more frequent for males than for females, and involved only animals of two or more years old (Additional File 1: Table S1).

### Group size and structure

The mean group size was 7.58 ± 8.90 individuals (ranged from 1 to 32; n = 48) and did not differ between years (F(1, 41) = 0.062; *P* = 0.804), or seasons (F(1, 41) = 0.009; *P* = 0.925). The composition of the groups are shown in Table 2. The mixed herds were the most common (23 herds) and significantly larger than the other group types (F(4, 41) = 6.311; *P* = 0.0005).

**Table 2:** Composition of the Western Derby eland herds recorded in the Niokolo Koba National Park, according to the defined age and sex categories, in both 2017 and 2018.

Age–sex category	Abbreviation in text	2017		2018	
		Total number (number of ID R)	Mean number per event (range)	Total number (number of ID R)	Mean number per event (range)
Adult males	ADM	12 (1)	0.75 (0–2)	30 (11)	0.94 (0–5)
Adult females	ADF	17 (2)	1.06 (0–3)	32 (17)	1.00 (0–5)
Adults unidentified	ADU	23 (0)	1.44 (0–7)	12 (0)	0.38 (0–4)
2-year old males	2YM	7 (1)	0.44 (0–2)	15 (4)	0.47 (0–4)
2-year old females	2YF	5 (0)	0.31 (0–3)	2 (2)	0.06 (0–1)
2-year olds unidentified	2YU	13 (0)	0.81 (0–4)	8 (0)	0.25 (0–3)
1-year old males	1YM	4 (0)	0.25 (0–2)	8 (0)	0.25 (0–2)
1-year old females	1YF	1 (1)	0.06 (0–1)	6 (2)	0.19 (0–3)
1-year old unidentified	1YU	5 (0)	0.31 (0–2)	8 (1)	0.25 (0–4)
Juveniles	JUV	32 (1)	2.00 (0–9)	48 (2)	1.50 (0–9)
Unknown at all	UNK	40 (0)	2.50 (0–13)	36 (1)	1.13 (0–6)
Total		159 (6)	9.94 (1–32)	205 (40)	6.41 (1–32)

The total numbers represent the total number of animals recorded, and therefore the absolute numbers are not directly comparable between years. ‘ID R’ refers to the individually recognized (identified) animals.

There were 16 solitary individuals recorded, of which five were ADMs, and one 2YM, while the rest were not identified. Out of the remaining 32 events with multiple individuals in the herd, only three contained the non-identifiable individuals (between 3 and 6 animals).

### Vital rate parameters

In 2017, the overall sex ratio was completely balanced (1:1), while the adult sex ratio was female-biased, specifically being 0.7:1. Juveniles formed 21% of all observed individuals, 23% were subadults, and 40% were adults (Table 2). The remaining 34 individuals (22%) could not be assigned to any age-sex category. The breeding rate derived from the accessible data in 2017 was high, and potentially approaching 100%, considering that there were more calves recorded (32 JUV) than adult females (31 ADF, considering the adult sex ratio). The annual calf survival rate was 31% (32 JUV and 10 1Y), while it sharply increased between the 1Y and 2Y in 2017 (10 1Y and 25 2Y). The annual survival rate was lower for ADM (64%) than for ADF (85%).

The overall sex ratio in 2018 was male dominated, being 1.3:1. On the contrary, the adult sex ratio was slightly female-biased, at 0.9:1. Juveniles formed 24% of all observed individuals, while 23% were subadults, and 36% were adults (Table 3). The remaining 36 individuals (18%) could not be assigned to any age-sex category. The breeding rate derived from the accessible data in 2018 was also high, and potentially approaching 100%, considering that there were more calves recorded (48 JUV) than adult females (37 ADF, considering the adult sex ratio). The annual survival rate derived from the data was 46% for JUV (48 JUV and 22 1Y), while it increased for the 1Y and 2Y in 2018 (22 1Y and 25 2Y). The annual survival was lower for ADM (61%) than for ADF (82%).

**Table 3:** Comparison of the Derby eland population structure, showing the ratio of juvenile (JUV), 1-year (1Y) and 2-year (2Y) olds, and adult (AD, ˃ 2 years old) individuals in the fenced reserves in Senegal, as calculated from Studbook 2008 (Antonínová et al.^33^) and Studbook 2019 (Brandlová et al.^52^).

	Studbook 2008	Studbook 2019	Chinko 2017*	Niokolo 2017*	Niokolo 2018*
% (N) JUV	16 (8)	8 (9)	15 (16)	27 (32)	29 (48)
% (N) 1Y	21 (10)	10 (12)		8 (10)	13 (22)
% (N) 2Y	16 (8)	13 (15)	25 (26)**	21 (25)	15 (25)
% (N) AD	47 (23)	63 (89)	59 (61)	44 (52)	43 (72)
Total population	100 (49)	100 (118)	100 (103)	100 (119)	100 (168)
Growth rate ƛ	1.38	1.12			

The number of recorded detections in the Chinko Protected Area, Central African Republic (Brandlová et al.^13^), and the current datasets are reported, and referred to as ‘Niokolo 2017’ and ‘Niokolo 2018’. The growth rates were calculated for the population in the fenced reserves, and show high values, even if a much lower number of JUV and SUB individuals are reported than for that of the wild populations.

*Numbers of animals detected using the camera traps serve only as indexes of population size.

**The number contains the sum of 1Y and 2Y individuals.

Most of the young calves (up to four months old) were recorded in January and February in both years, and the horns reached the ear length in April, which suggests that the peak of the breeding period occurs in November and December, with few calves potentially born in January.

### Population size

Density estimate (± SE), based on the best-fitting model (hazard rate, AICc = 563.489) for 30 individually identified animals in 38 detections, was 0.138 WDE/km^2^ (± 0.0102). g0 was estimated at 0.001 (SE ± 0.001) and sigma at 4092 (SE ± 301). Given the mask area calculated as 1413 km^2^, the population size of the WDE in NKNP can be estimated as 195 individuals, in confidence limits (95%) ranging from 54 to 708 individuals.

## Discussion

Based on the extensive camera trap study, the very first information about the occupancy, trapping rate, activity pattern, group size, social structure and vital rates of the critically endangered Western Derby eland in its last refugium, the NKNP in Senegal, is presented here. The first estimation of abundance since 2006 is also provided^7^.

### Spatiotemporal behaviour pattern of WDE in the park

The results of the CT survey in the NKNP highlight the substantially lower occupancy and trapping rate of WDE in comparison to other large ungulates in the park. According to the current results, the WDE occupied less than 5% of the park area during the dry season, being exclusively within the zone of Mont Assirik, and more specifically the Mansa Fara marsh, which can be thus designated as the core area of the WDE distribution. The trapping rate of the roan antelope, which is considered the most abundant antelope species in the NKNP, was 4.04, i.e. more than 11 times higher than that of WDE in the present study. Even the Western hartebeest, which is considered a rare species in the NKNP, had a trapping rate of 0.61, which is ca. twice as high that that of the WDE (see Rabeil et al.^8^ for further details, and additional ungulate species).

In the zone of Mont Assirik, the trapping rate of WDE increased to 2.42, but the trapping rates of other antelope species remained higher still (4.6 for roan antelope and 3.39 for Western hartebeest^8^). The WDE distribution is therefore strongly localised within an area which seems to also be attractive for the other species, including the incidental records of elephant. The Mont Assirik zone, and more specifically the Mansa Fara marsh area, is therefore the crucial zone within the park for the WDE, and it appears to support a larger number of other antelope species as well. This zone should therefore be considered as a key conservation area, potentially very sensitive to targeted poaching, and thus crucial for efficacy of targeted law enforcement actions.

When looking at the diurnal activity pattern, the WDE were active before midnight, approximately 3 h after sunset, in the morning, approximately 2 h after sunrise, and then again in the afternoon, with the peak activity during the hottest part of the day. This activity pattern is different from the typical bimodal activity pattern, which has peaks at dawn and dusk, as reported for most African grazing and browsing herbivores, seen as a behavioural thermoregulation strategy to avoid heat stress^41–43^. Instead, the WDE, being a large body-sized browsing antelope^19,44^, must stay active throughout the day to seek discretely distributed food, and fulfil foraging requirements by feeding while moving. The WDE appears to be well-adapted to tolerate such high temperatures, similar to kudu^45^, roan^46^, and giraffe^47^. Such behaviour pattern enable the law enforcement patrols, as well to tourists, to detect herds of WDEs and monitor them, thereby increasing their protection against poaching.

### Individual identification and recaptures

The individual identification of animals was more successful during the daytime, as the light conditions mostly did not allow for the proper visualization of the stripes on the flanks during the night captures (as similarly reported in Jůnek et al.^18^). When the ID is targeted to be successful during the night (as for the leopards and tigers), the camera traps are often set to the video mode to ensure a higher possibility of identification^48^. However, the activity of the WDE is not predominantly nocturnal, and the captures were distributed over both the daylight and night hours, and therefore the results are considered representative for the whole period.

The AD animals were more likely to be identified in the present study because of their larger body size, resulting in better visibility of their stripes. The higher identification rate of larger individuals also likely contributed to the higher probability of recaptures, which were only recorded for individuals of 2Y and older.

Overall, the identification success rate was comparable, maybe even slightly higher, than the previous camera trap study performed on the Eastern Giant eland in Chinko, CAR, specifically in the dataset from the dry season^13^, which corresponds to the observation period in the present study as well.

In the NKNP, recaptures of individuals were recorded, whereas there were none reported in Chinko^13^. The recapture rate of the WDE in NKNP, with mostly short distances between the capture-recapture sites, even after the long-time gaps between the captures, confirm again that the WDE likely inhabit a relatively limited area of the park.

### Group size and social structure

The mean group size recorded in the NKNP during the present study was slightly larger than that within Chinko; however, the maximum group size was smaller in NKNP (32 vs. 41 individuals). Mixed herds were the largest in terms of the number of individuals, in both studies. The average group size has been reported as 20–30 individuals^49^, but Derby elands may form large herds of over 100 individuals in the late dry season^14^. Similarly, a large herd was reported within NKNP in 2006, having 69 individuals^7^, and a herd of around 60 WDE was also recently reported by patrols in 2020 (GIE Niokolo, personal communications). It is important to highlight that the results from the present study reflect the number of individuals per event based on visible individuals within the scope of the camera, and that the real group sizes may actually be larger.

No adult males were present in the mixed herd in two cases within the present study; however, there were always 2YM and a few unidentified individuals, suggesting that the herd should not be considered as a pure “nursery herd”, as known for sexually dimorphic antelope species^50^.

Calves are born in the NKNP during the period comparable to that of Bandia, Fathala and Chinko, i.e. during the early dry season^16^. The higher proportion of calves in the dry season corresponds with the nursing period of six months for WDE^44^. Given a pregnancy length of nine months, the WDE mating season in NKNP peaks in January/February, which also corresponds with the formation of large herds with multiple males, as similarly seen in Chinko and Cameroon^13,14^.

### Vital rates

The sex ratio of the WDE in the NKNP was female-biased. The skewed adult sex ratio reflects the lower survival rate of males in comparison with females, typical for polygynous species^51^. This result also corresponds with the findings from other Derby eland populations, namely from Chinko, where the bias towards females in the adult sex ratio was even more pronounced (0.67:1^13^). A similar ratio was found in the hunting reserves within Cameroon^35^, but also in the semi-captive population, without hunting and without predators^34^. As the ratio in NKNP was less skewed than that within Chinko and Cameroon, a lower or zero selectivity for males by hunters/poachers is expected.

The population of WDE in the NKNP showed a lower proportion of adults versus other age categories compared to the demographic structure of the WDE in the semi-captive breeding facilities of the Bandia and Fathala reserve^33,52^, and to those of the Eastern subspecies of Derby eland in the Central African Republic^13^ (see Table 3). The data from the present study also showed a surprisingly high breeding rate (likely close to 100%), as well as a high survival rate of yearlings. This combination of demographic characteristics should be highly favourable, and likely to lead to a significant population growth rate; however, this does not seem to be the case of the WDE population in the NKNP (please refer to further discussion about population size).

In this context, the population of WDE in the NKNP was explored deeper, to examine possible scenarios of changes within the population structure. The changes in vital rates between two years of monitoring (2017 and 2018) were examined, by taking advantage of the possible recognition of the age category until two years of age, and the knowledge of the life tables of the enclosed, non-predated WDE population in the Bandia reserve^34^. Life tables were created for each year, and for males (M) and females (F) separately, according to the standard structure^2^, and based on two scenarios: a) only the observed number of JUV and 1Y (nx), and modelled 2Y (model ‘JUV + 1Y’); b) the observed number of JUV and 2Y (nx) (model ‘JUV + 2Y’). Then, estimations of animals in age categories based on two parameters were calculated: (i) based on the mortality rate (qx) known from the Bandia reserve (Senegal), and (ii) based on the recorded number of animals (N_AD_), to calculate the estimation of mortality rate (for details, see Additional file 1: Table S2).

The resulting values demonstrated that with survival rates comparable to a population without predation and poaching, the number of adults would be twice or three times higher than currently detected in the present study. Yet, considering the recorded number of adult individuals, the annual adult survival rate was considerably low, i.e. 59–69% in males and 67–82% for females. To conclude, the demographic structure of WDE in NKNP showed a high breeding rate, moderate juvenile survival, high survival rate of yearlings, and a low survival rate of adults.

Juvenile survival is one of the most fluctuating vital rate parameters, sensitive to population density, stochastic environmental variation, and predation^53–55^. Given the high proportion of juveniles within the population, and the breeding rate higher than that in Cameroon (74%^14^) and within the captive population (77%^34^), the juvenile survival rate does not seem to negatively affect the population growth in the NKNP. High breeding rates could be a more robust determinant of population change than AD mortality^53^, and it is therefore possible that the WDE population size is stable in the NKNP, or even increasing, despite the low adult survival rates. On the other hand, the relatively low numbers of AD individuals in the population indicates low survival rates, which may lead to the decline and final crash of the population^54^. It is acknowledged that data from two consecutive years was used in the present study, which were not comparable due to different CT settings, and that long-term monitoring, which accounts for variability in vital rates, would be a conservation essential to identify the trend and population change.

Based on the present findings of WDE spatiotemporal behaviour and estimates of vital rates, several explanations about multiple processes interacting in the environmental, anthropogenic and conservation context of the park, which inherently affect the small population of WDE, can be inferred. One explanation may suggest that a low proportion of AD WDE and higher JUV survival rates may reflect the influence of growing populations of apex predators in the NKNP, specifically the population of lions^56^, which may preferentially target the adult individuals^57^. The age-sex structure also encourages the interpretation that the adult animals are exposed to human-related factors, which prevents them from expanding from the core area of their distribution, exacerbating male-male competition in the limited space^34^. The poaching activity was also highlighted as an existing threat to WDE populations^35^. However, law enforcement has been substantially intensified in the core and south-eastern part of the NKNP since 2017^58^, and lion-conservation actions are specifically supported. Thus, the predator populations may have started to grow, which is confirmed by the relative high trapping rate of lions in this core area^8^. Hence, increased predation may interfere with other environmental factors and consequently affect the WDE population dynamics at the level of AD individuals^55,59^.

A complementary scenario may highlight other factors, specifically, those which maintain the WDE population within a certain spatial extent of the park, i.e. Mont Assirik and Mansa Fara marsh zone. This area can be delimited either ecologically by specific unidentified resources, or by anthropogenic factors, namely a highly frequented trade road crossing the park, wild bushfires, and intensive livestock encroachment in a large band from the borders of the park, inwards (up to 10 km). There is also a vast area in the central part of the park that offers an important space with a supposed carrying capacity for large herbivore populations. This area is, however, outside of the zone of intensified law enforcement, and suffers from inadequate surveillance in the long-term, due to the absence of tracks and therefore being difficult for rangers to access. This area certainly represents an attractive zone for targeted illegal hunting actions. These limiting factors constrain large mammals to concentrate within the zone of Mount Assirik and Mansa Fara marsh, which, in turn, makes animal populations vulnerable to any potential environmental or man-induced incidents, like bush fire.

### Population size

The estimated population size of 195 individuals corresponds with the range of most recent estimates of the WDE population size in the NKNP, i.e. 100–200 (approximately 170) individuals^6,7,60^. Given the fact that the model contains only the data for AD animals (as no other age category had recapture records), it may be considered that this estimate refers to the number of adult individuals in the population. With regards to Table 3, showing that adults are likely to form 43 to 44% of the whole population, it may be inferred that the actual number of WDE in the NKNP could be higher, even up to 300 individuals, if the data are corrected for the 22% of unidentified individuals. The WDE density estimate of 0.138 individuals/km^2^ was comparable to densities of Eastern Derby eland in CAR (densities ranging between 0.04 and 0.16 individuals/km^2^), in Chinko^13^, and ranging between 0.002 and 0.1 individuals/km^2^ in the northern CAR^61^, as well as in Cameroon, with densities ranging between 0.002 and 0.08 individuals/km^2^^62^. On the other hand, in comparison to other antelope species, the estimated WDE density falls within the range of densities of large herbivores reported from many other sites in African protected areas^63^, where lower values correspond to the larger areas and are also associated with large browsers, i.e. to the type of diet. Maximum densities of a healthy undisturbed DE population were estimated at about 0.5 individuals/km^2^^49^, and can reach up to 1.19 individuals/km^2^ in intensively surveyed hunting zones in Northern CAR^61^. Thus, the density of WDE in the NKNP could be potentially higher.

## Conclusion

Findings highlighted that the critically endangered Western Derby eland population has the potential of growing dynamics in the NKNP, with an underutilized capacity for population density. The age-specific vital rates indicate that the most vulnerable element in the population is the survival of adult individuals, which suggests a potential effect of an increased large predator population, or a man-induced issue connected with poaching, livestock encroachment, and/or bushfires in the NKNP. The clear spatiotemporal behaviour pattern of WDE in the NKNP, i.e. the occurrence of WDE within a specific zone of the park and the possibility to observe the herds during the daylight, enables targeted monitoring and protection by the protected area management decision-makers. Understanding of the vital rates, habitat (resource) selection, and movement ecology of the WDE is required, to bring critical insights for science-based conservation of the WDE’s small population. It is therefore recommended that a detailed study of the space and resources use by WDE in the NKNP should be conducted, preferentially by the means of satellite collars, which may bring further insight into the limitations of WDE population size and distribution.

## Supplementary Information

Supplementary Information.

## Data Availability

The datasets used and/or analyzed during the current study are available from the corresponding authors on reasonable request.
