# Supplementary material for: Spatially restricted occurrence and low abundance as key tools for conservation of critically endangered large antelope in West African savannah
Source: Sci Rep. 2021 Sep 29;11:19397. doi: 10.1038/s41598-021-98649-7 (PMC8481223; doi:10.1038/s41598-021-98649-7)
Supplement: Supplementary file 1 — Supplementary Information. [file 41598_2021_98649_MOESM1_ESM.docx]

**Additional file 1**

**Table S1.** The list of individual animals captured from either left (L) or right (R) side with dates of capture and recapture and distances between recaptures.

| **Individual** | **Age-sex** | **CTstat** | **Year** | **Date** | **Time (days)** | **Distance (m)** |
| --- | --- | --- | --- | --- | --- | --- |
| 10R | 2Y_M | Mont3 | 2018 | 20/01/2018 |  |  |
| 10R | 2Y_M | Mont3 | 2018 | 22/01/2018 | 2 | 0 |
| 8L | 2Y_M | Mont3 | 2018 | 20/01/2018 |  |  |
| 8L | 2Y_M | Mont3 | 2018 | 22/01/2018 | 2 | 0 |
| 8L | 2Y_M | Mont3 | 2018 | 05/02/2018 | 14 | 0 |
| 13R | AD_F | Mont1 | 2018 | 20/01/2018 |  |  |
| 13R | AD_F | Mont3 | 2018 | 28/01/2018 | 8 | 4735 |
| 13R | AD_F | Mont3 | 2018 | 28/04/2018 | 90 | 0 |
| 21R | 2Y_F | CT 2016 | 2016 | 02/03/2016 |  |  |
| 21R | AD_F | Mont3 | 2018 | 28/04/2018 | 787 | 1884 |
| 22R | AD_F | Mont11 | 2017 | 17/03/2017 |  |  |
| 22R | AD_F | Mont3 | 2018 | 28/04/2018 | 407 | 1884 |
| 28R | AD_M | Mont3 | 2018 | 20/01/2018 |  |  |
| 28R | AD_M | Mont3 | 2018 | 22/01/2018 | 2 | 0 |
| 28R | AD_M | Mont3 | 2018 | 28/01/2018 | 6 | 0 |
| 28R | AD_M | Mont3 | 2018 | 05/02/2018 | 8 | 0 |
| 30R | AD_M | Mont11 | 2017 | 04/04/2017 |  |  |
| 30R | AD_M | Mont3 | 2018 | 25/01/2018 | 296 | 1884 |
| 30R | AD_M | Mont3 | 2018 | 28/01/2018 | 3 | 0 |
| 30R | AD_M | Mont3 | 2018 | 30/01/2018 | 2.2 | 0 |
| 12L | AD_F | Mont4b | 2018 | 29/04/2018 | 97 | 1656 |
| 12L | AD_F | Mont3 | 2018 | 22/01/2018 |  |  |
| 25L | AD_M | Mont7 | 2017 | 06/04/2017 |  |  |
| 25L | AD_M | Mont3 | 2018 | 31/01/2018 | 300 | 1650 |
| 26L | AD_M | Mont3 | 2018 | 31/01/2018 |  |  |
| 26L | AD_M | Mont3 | 2018 | 05/02/2018 | 5 | 0 |

**Table S2.** Modelled scenarios of life tables for Western Derby eland population in the Niokolo Koba national park in Senegal in 2018 and 2017. To correct for the bias caused by higher detected numbers of 2Y than 1Y individuals (which would result in negative q1 values), two different scenarios were used as follows: a) only the observed number of JUV and 1Y (nx) and modelled 2Y; b) the observed number of JUV and 2Y (nx), and truncated the lx 1 to 1 as in Johnson et al (2010). Life tables were modelled separately for males and females, for 2018 and 2017, using separately data on numbers of 1-year (model ‘JUV+1Y ‘) and 2-years (model ‘JUV+2Y ‘) old animals, and calculated using two approaches: one is the estimation of animals in age categories based on the mortality rate (qx) known from enclosed, non-predated population in the Bandia reserve (Senegal), and the second approach is based on the recorded number of animals (N_AD)_ to calculate the estimation of mortality rate.

The mean annual mortalities from modelled data were calculated as follows:

M_anADF_ = mean Fqx for Fnx>2

M_anADM_ = mean Mqx for Mnx>2

Life tables of both scenarios showed the potential numbers of AD individuals in the population in case the mortalities were reduced to those reported from fenced areas. According to the first scenario (JUV+1Y), the number of potential detections of ADM was 47 and those of ADF was 34, which represent 156% of the detected individuals in 2017 and 80 ADM and 67 ADF representing 201 % of the current number of detections in 2018 (Table S2: a-d). According to the second scenario (JUV+2Y) where the JUV mortality was significantly reduced by adjusting the number of detections of 1Y individuals to this of 2Y individuals, the number of potential detections was even higher. 91 for ADM and 84 for ADF, which represent 337% of the detected individuals in 2017 and 133 ADM and 42 ADF representing 240 % of the current number of detections in 2018 (Table S2: e-h).

Mortality rates derived from life tables using the observed number of detections is shown in Table S2e – p. According to the first scenario (JUV+1Y), MadM was 31% and MadF was 18% in 2017, and 33% and 27% for 2018 respectively (Table S2: e – h). According to the second scenario (JUV+2Y), MadM was 39% and MadF was 36% in 2017, and 41% and 20% for 2018 respectively (Table S2: m – p). In the second scenario, the adjustment of 1Y number of detections resulted in following JUV mortalities: MJUVM = 19%, MJUVF = 25% in 2017 and MJUVM = 20% and MJUVF = 58% in 2018.

***Abbreviations:*** x – age class of animals (in years, ranging 0 – x), nx – number of detected individuals alive at start of given age class, lx – proportion of animals surviving at start of given age interval to initial number (relative number ranging 0 – 1), dx – number of individuals dying within age interval x to x+1, qx - finite rate of mortality within the interval (relative number ranging 0 – 1), px - finite rate of survival (relative number ranging 0 – 1).

**Tables S2 a – d, i – l** : Estimation of nx (indicated by black bold) based on the known mortality rate of each age class in the Bandia reserve (qx indicated by green italics). Real observed numbers of animals are indicated by red bold.

**Table S2 a.** The life table for males in 2018 using the model ‘JUV+1Y’ for estimation of nx.

| **x (years)** | **nx** | **lx** | **dx** | **qx** | **px** |
| --- | --- | --- | --- | --- | --- |
| 0 | **24** | 1.00 | 12.00 | 0.50 | 0.50 |
| 1 | **12** | 0.50 | 0.50 | ***0.04*** | 0.96 |
| 2 | **11** | 0.48 | 0.68 | ***0.06*** | 0.94 |
| 3 | **11** | 0.45 | 0.28 | ***0.03*** | 0.97 |
| 4 | **11** | 0.44 | 0.63 | ***0.06*** | 0.94 |
| 5 | **10** | 0.41 | 0.54 | ***0.06*** | 0.95 |
| 6 | **9** | 0.39 | 0.62 | ***0.07*** | 0.93 |
| 7 | **9** | 0.36 | 0.47 | ***0.05*** | 0.95 |
| 8 | **8** | 0.34 | 1.12 | ***0.14*** | 0.87 |
| 9 | **7** | 0.30 | 1.18 | ***0.17*** | 0.84 |
| 10 | **6** | 0.25 | 0.85 | ***0.14*** | 0.86 |
| 11 | **5** | 0.21 | 2.20 | ***0.43*** | 0.57 |
| 12 | **3** | 0.12 | 1.46 | ***0.50*** | 0.50 |
| 13 | **1** | 0.06 | 1.22 | ***0.83*** | 0.17 |
| 14 | **0** |  |  |  |  |

**Table S2 b.** The life table for females in 2018 using the model ‘JUV+1Y’ for estimation of nx.

| **x (years)** | **nx** | **lx** | **dx** | **qx** | **px** |
| --- | --- | --- | --- | --- | --- |
| 0 | **24** | 1.00 | 14.00 | 0.58 | 0.42 |
| 1 | **10** | 0.42 | 0.42 | ***0.04*** | 0.96 |
| 2 | **10** | 0.40 | 0.57 | ***0.06*** | 0.94 |
| 3 | **9** | 0.38 | 0.23 | ***0.03*** | 0.97 |
| 4 | **9** | 0.37 | 0.53 | ***0.06*** | 0.94 |
| 5 | **8** | 0.34 | 0.45 | ***0.06*** | 0.95 |
| 6 | **8** | 0.32 | 0.51 | ***0.07*** | 0.93 |
| 7 | **7** | 0.30 | 0.39 | ***0.05*** | 0.95 |
| 8 | **7** | 0.29 | 0.93 | ***0.14*** | 0.87 |
| 9 | **6** | 0.25 | 0.98 | ***0.17*** | 0.84 |
| 10 | **5** | 0.21 | 0.71 | ***0.14*** | 0.86 |
| 11 | **4** | 0.18 | 1.83 | ***0.43*** | 0.57 |
| 12 | **2** | 0.10 | 1.22 | ***0.50*** | 0.50 |
| 13 | **1** | 0.05 | 1.01 | ***0.83*** | 0.17 |
| 14 | **0** |  |  |  |  |

**Table S2 c.** The life table for males in 2017 using the model ‘JUV+1Y’ for estimation of nx.

| **x (years)** | **nx** | **lx** | **dx** | **qx** | **px** |
| --- | --- | --- | --- | --- | --- |
| 0 | **16** | 1.00 | 9.00 | 0.56 | 0.44 |
| 1 | **7** | 0.44 | 0.29 | ***0.04*** | 0.96 |
| 2 | **7** | 0.42 | 0.40 | ***0.06*** | 0.94 |
| 3 | **6** | 0.39 | 0.16 | ***0.03*** | 0.97 |
| 4 | **6** | 0.38 | 0.37 | ***0.06*** | 0.94 |
| 5 | **6** | 0.36 | 0.32 | ***0.06*** | 0.95 |
| 6 | **5** | 0.34 | 0.36 | ***0.07*** | 0.93 |
| 7 | **5** | 0.32 | 0.28 | ***0.05*** | 0.95 |
| 8 | **5** | 0.30 | 0.65 | ***0.14*** | 0.87 |
| 9 | **4** | 0.26 | 0.69 | ***0.17*** | 0.84 |
| 10 | **3** | 0.22 | 0.50 | ***0.14*** | 0.86 |
| 11 | **3** | 0.19 | 1.28 | ***0.43*** | 0.57 |
| 12 | **2** | 0.11 | 0.85 | ***0.50*** | 0.50 |
| 13 | **1** | 0.05 | 0.71 | ***0.83*** | 0.17 |
| 14 | **0** |  |  |  |  |

**Table S2 d.** The life table for females in 2017 using the model ‘JUV+1Y’ for estimation of nx.

| **x (years)** | **nx** | **lx** | **dx** | **qx** | **px** |
| --- | --- | --- | --- | --- | --- |
| 0 | **16** | 1.00 | 11.00 | 0.69 | 0.31 |
| 1 | **5** | 0.31 | 0.21 | ***0.04*** | 0.96 |
| 2 | **5** | 0.30 | 0.28 | ***0.06*** | 0.94 |
| 3 | **5** | 0.28 | 0.12 | ***0.03*** | 0.97 |
| 4 | **4** | 0.27 | 0.26 | ***0.06*** | 0.94 |
| 5 | **4** | 0.26 | 0.23 | ***0.06*** | 0.95 |
| 6 | **4** | 0.24 | 0.26 | ***0.07*** | 0.93 |
| 7 | **4** | 0.23 | 0.20 | ***0.05*** | 0.95 |
| 8 | **3** | 0.22 | 0.47 | ***0.14*** | 0.87 |
| 9 | **3** | 0.19 | 0.49 | ***0.17*** | 0.84 |
| 10 | **2** | 0.16 | 0.36 | ***0.14*** | 0.86 |
| 11 | **2** | 0.13 | 0.92 | ***0.43*** | 0.57 |
| 12 | **1** | 0.08 | 0.61 | ***0.50*** | 0.50 |
| 13 | **1** | 0.04 | 0.51 | ***0.83*** | 0.17 |
| 14 | **0** |  |  |  |  |

**Tables S2 e – h, m – p**: Estimation of qx (indicated by black bold) based on the observed numbers of animals in x = 0 and x= 1. The next nx (indicated by green italics) represents recorded N_AD_  distributed into age classes x to fit the modelled qx values. Real observed numbers of animals are indicated by red bold.

**Table S2 e.** The life table for males in 2018 using the model ‘JUV+1Y’ for estimation of qx.

| **x (years)** | **nx** | **lx** | **dx** | **qx** | **px** |
| --- | --- | --- | --- | --- | --- |
| 0 | **24** | 1.00 | 12.00 | 0.50 | 0.50 |
| 1 | **12** | 0.50 | 1.20 | **0.10** | 0.90 |
| 2 | ***11*** | 0.45 | 1.08 | **0.10** | 0.90 |
| 3 | ***10*** | 0.41 | 1.94 | **0.20** | 0.80 |
| 4 | ***8*** | 0.32 | 1.56 | **0.20** | 0.80 |
| 5 | ***6*** | 0.26 | 1.56 | **0.25** | 0.75 |
| 6 | ***5*** | 0.19 | 1.40 | **0.30** | 0.70 |
| 7 | ***3*** | 0.14 | 1.31 | **0.40** | 0.60 |
| 8 | ***2*** | 0.08 | 0.78 | **0.40** | 0.60 |
| 9 | ***1*** | 0.05 | 0.94 | **0.80** | 0.20 |
| 10 | ***0*** |  |  |  |  |
| 11 | ***0*** |  |  |  |  |
| 12 | ***0*** |  |  |  |  |
| 13 | ***0*** |  |  |  |  |
| 14 | ***0*** |  |  |  |  |

**Table S2 f.** The life table for females in 2018 using the model ‘JUV+1Y’ for estimation of qx.

| **x (years)** | **nx** | **lx** | **dx** | **qx** | **px** |
| --- | --- | --- | --- | --- | --- |
| 0 | **24** | 1.00 | 14.00 | 0.58 | 0.42 |
| 1 | **10** | 0.42 | 1.00 | **0.10** | 0.90 |
| 2 | ***9*** | 0.38 | 0.90 | **0.10** | 0.90 |
| 3 | ***8*** | 0.34 | 0.81 | **0.10** | 0.90 |
| 4 | ***7*** | 0.30 | 0.73 | **0.10** | 0.90 |
| 5 | ***7*** | 0.27 | 1.31 | **0.20** | 0.80 |
| 6 | ***5*** | 0.22 | 1.05 | **0.20** | 0.80 |
| 7 | ***4*** | 0.17 | 0.84 | **0.20** | 0.80 |
| 8 | ***3*** | 0.14 | 1.01 | **0.30** | 0.70 |
| 9 | ***2*** | 0.10 | 0.94 | **0.40** | 0.60 |
| 10 | ***1*** | 0.06 | 1.13 | **0.80** | 0.20 |
| 11 | ***0*** |  |  |  |  |
| 12 | ***0*** |  |  |  |  |
| 13 | ***0*** |  |  |  |  |
| 14 | ***0*** |  |  |  |  |

**Table S2 g.** The life table for males in 2017 using the model ‘JUV+1Y’ for estimation of qx.

| **x (years)** | **nx** | **lx** | **dx** | **qx** | **px** |
| --- | --- | --- | --- | --- | --- |
| 0 | **16** | 1.00 | 9.00 | 0.56 | 0.44 |
| 1 | **7** | 0.44 | 0.70 | **0.10** | 0.90 |
| 2 | ***6*** | 0.39 | 0.63 | **0.10** | 0.90 |
| 3 | ***6*** | 0.35 | 0.57 | **0.10** | 0.90 |
| 4 | ***5*** | 0.32 | 1.02 | **0.20** | 0.80 |
| 5 | ***4*** | 0.26 | 0.82 | **0.20** | 0.80 |
| 6 | ***3*** | 0.20 | 0.98 | **0.30** | 0.70 |
| 7 | ***2*** | 0.14 | 0.91 | **0.40** | 0.60 |
| 8 | ***1*** | 0.09 | 0.55 | **0.40** | 0.60 |
| 9 | ***1*** | 0.05 | 0.66 | **0.80** | 0.20 |
| 10 | ***0*** |  |  |  |  |
| 11 | ***0*** |  |  |  |  |
| 12 | ***0*** |  |  |  |  |
| 13 | ***0*** |  |  |  |  |
| 14 | ***0*** |  |  |  |  |

**Table S2 h.** The life table for females in 2017 using the model ‘JUV+1Y’ for estimation of qx.

| **x (years)** | **nx** | **lx** | **dx** | **qx** | **px** |
| --- | --- | --- | --- | --- | --- |
| 0 | **16** | 1.00 | 11.00 | 0.69 | 0.31 |
| 1 | **5** | 0.31 | 0.50 | **0.10** | 0.90 |
| 2 | ***5*** | 0.28 | 0.45 | **0.10** | 0.90 |
| 3 | ***4*** | 0.25 | 0.41 | **0.10** | 0.90 |
| 4 | ***4*** | 0.23 | 0.36 | **0.10** | 0.90 |
| 5 | ***3*** | 0.21 | 0.33 | **0.10** | 0.90 |
| 6 | ***3*** | 0.18 | 0.30 | **0.10** | 0.90 |
| 7 | ***3*** | 0.17 | 0.27 | **0.10** | 0.90 |
| 8 | ***2*** | 0.15 | 0.24 | **0.10** | 0.90 |
| 9 | ***2*** | 0.13 | 0.22 | **0.10** | 0.90 |
| 10 | ***2*** | 0.12 | 0.19 | **0.10** | 0.90 |
| 11 | ***2*** | 0.11 | 0.35 | **0.20** | 0.80 |
| 12 | ***1*** | 0.09 | 0.56 | **0.40** | 0.60 |
| 13 | ***1*** | 0.05 | 0.33 | **0.40** | 0.60 |
| 14 | ***1*** | 0.03 | 0.20 | **0.40** | 0.60 |
| 15 | ***0*** |  |  |  |  |

**Table S2 i.** The life table for males in 2018 using the model ‘JUV+2Y’ for estimation of nx.

| **x (years)** | **nx** | **lx** | **dx** | **qx** | **px** |
| --- | --- | --- | --- | --- | --- |
| 0 | **24** | 1.00 | 5.00 | 0.21 | 0.79 |
| 1 | **19** | 0.79 | 0.80 | ***0.04*** | 0.96 |
| 2 | **19** | 0.79 | 1.12 | ***0.06*** | 0.94 |
| 3 | **18** | 0.74 | 0.46 | ***0.03*** | 0.97 |
| 4 | **17** | 0.73 | 1.04 | ***0.06*** | 0.94 |
| 5 | **16** | 0.68 | 0.90 | ***0.06*** | 0.95 |
| 6 | **15** | 0.64 | 1.02 | ***0.07*** | 0.93 |
| 7 | **14** | 0.60 | 0.78 | ***0.05*** | 0.95 |
| 8 | **14** | 0.57 | 1.85 | ***0.14*** | 0.87 |
| 9 | **12** | 0.49 | 1.95 | ***0.17*** | 0.84 |
| 10 | **10** | 0.41 | 1.41 | ***0.14*** | 0.86 |
| 11 | **8** | 0.35 | 3.63 | ***0.43*** | 0.57 |
| 12 | **5** | 0.20 | 2.42 | ***0.50*** | 0.50 |
| 13 | **2** | 0.10 | 2.01 | ***0.83*** | 0.17 |
| 14 | **0** |  |  |  |  |

**Table S2 j.** The life table for females in 2018 using the model ‘JUV+2Y’ for estimation of nx.

| **x (years)** | **nx** | **lx** | **dx** | **qx** | **px** |
| --- | --- | --- | --- | --- | --- |
| 0 | **24** | 1.00 | 14.00 | 0.58 | 0.42 |
| 1 | **10** | 0.42 | 0.42 | ***0.04*** | 0.96 |
| 2 | **6** | 0.25 | 0.35 | ***0.06*** | 0.94 |
| 3 | **6** | 0.24 | 0.15 | ***0.03*** | 0.97 |
| 4 | **5** | 0.23 | 0.33 | ***0.06*** | 0.94 |
| 5 | **5** | 0.22 | 0.28 | ***0.06*** | 0.95 |
| 6 | **5** | 0.20 | 0.32 | ***0.07*** | 0.93 |
| 7 | **5** | 0.19 | 0.25 | ***0.05*** | 0.95 |
| 8 | **4** | 0.18 | 0.58 | ***0.14*** | 0.87 |
| 9 | **4** | 0.16 | 0.62 | ***0.17*** | 0.84 |
| 10 | **3** | 0.13 | 0.45 | ***0.14*** | 0.86 |
| 11 | **3** | 0.11 | 1.15 | ***0.43*** | 0.57 |
| 12 | **2** | 0.06 | 0.76 | ***0.50*** | 0.50 |
| 13 | **1** | 0.03 | 0.64 | ***0.83*** | 0.17 |
| 14 | **0** |  |  |  |  |

**Table S2 k.** The life table for males in 2017 using the model ‘JUV+2Y’ for estimation of nx.

| **x (years)** | **nx** | **lx** | **dx** | **qx** | **px** |
| --- | --- | --- | --- | --- | --- |
| 0 | **16** | 1.00 | 3.00 | 0.19 | 0.81 |
| 1 | **13** | 0.81 | 0.55 | ***0.04*** | 0.96 |
| 2 | **13** | 0.81 | 0.77 | ***0.06*** | 0.94 |
| 3 | **12** | 0.76 | 0.32 | ***0.03*** | 0.97 |
| 4 | **12** | 0.74 | 0.71 | ***0.06*** | 0.94 |
| 5 | **11** | 0.70 | 0.62 | ***0.06*** | 0.95 |
| 6 | **11** | 0.66 | 0.70 | ***0.07*** | 0.93 |
| 7 | **10** | 0.62 | 0.53 | ***0.05*** | 0.95 |
| 8 | **9** | 0.58 | 1.26 | ***0.14*** | 0.87 |
| 9 | **8** | 0.51 | 1.33 | ***0.17*** | 0.84 |
| 10 | **7** | 0.42 | 0.97 | ***0.14*** | 0.86 |
| 11 | **6** | 0.36 | 2.48 | ***0.43*** | 0.57 |
| 12 | **3** | 0.21 | 1.65 | ***0.50*** | 0.50 |
| 13 | **2** | 0.10 | 1.38 | ***0.83*** | 0.17 |
| 14 | **0** |  |  |  |  |

**Table S2 l.** The life table for females in 2017 using the model ‘JUV+2Y’ for estimation of nx.

| **x (years)** | **nx** | **lx** | **dx** | **qx** | **px** |
| --- | --- | --- | --- | --- | --- |
| 0 | **16** | 1.00 | 4.00 | 0.25 | 0.75 |
| 1 | **12** | 0.75 | 0.50 | ***0.04*** | 0.96 |
| 2 | **12** | 0.75 | 0.71 | ***0.06*** | 0.94 |
| 3 | **11** | 0.71 | 0.29 | ***0.03*** | 0.97 |
| 4 | **11** | 0.69 | 0.66 | ***0.06*** | 0.94 |
| 5 | **10** | 0.65 | 0.57 | ***0.06*** | 0.95 |
| 6 | **10** | 0.61 | 0.64 | ***0.07*** | 0.93 |
| 7 | **9** | 0.57 | 0.49 | ***0.05*** | 0.95 |
| 8 | **9** | 0.54 | 1.17 | ***0.14*** | 0.87 |
| 9 | **7** | 0.47 | 1.23 | ***0.17*** | 0.84 |
| 10 | **6** | 0.39 | 0.89 | ***0.14*** | 0.86 |
| 11 | **5** | 0.33 | 2.29 | ***0.43*** | 0.57 |
| 12 | **3** | 0.19 | 1.53 | ***0.50*** | 0.50 |
| 13 | **2** | 0.10 | 1.27 | ***0.83*** | 0.17 |
| 14 | **0** |  |  |  |  |

**Table S2 m.** The life table for males in 2018 using the model ‘JUV+2Y’ for estimation of qx.

| **x (years)** | **nx** | **lx** | **dx** | **qx** | **px** |
| --- | --- | --- | --- | --- | --- |
| 0 | **24** | 1.00 | 5.00 | 0.21 | 0.79 |
| 1 | **19** | 0.79 | 1.90 | **0.10** | 0.90 |
| 2 | **19** | 0.79 | 3.80 | **0.20** | 0.80 |
| 3 | ***15*** | 0.63 | 4.56 | **0.30** | 0.70 |
| 4 | ***11*** | 0.44 | 3.19 | **0.30** | 0.70 |
| 5 | ***7*** | 0.31 | 2.98 | **0.40** | 0.60 |
| 6 | ***4*** | 0.19 | 1.79 | **0.40** | 0.60 |
| 7 | ***3*** | 0.11 | 1.34 | **0.50** | 0.50 |
| 8 | ***1*** | 0.06 | 1.07 | **0.80** | 0.20 |
| 9 | ***0*** | 0.01 |  |  |  |
| 10 | ***0*** | 0.01 |  |  |  |
| 11 | ***0*** | 0.01 |  |  |  |
| 12 | ***0*** | 0.01 |  |  |  |
| 13 | ***0*** | 0.01 |  |  |  |
| 14 | ***0*** | 0.01 |  |  |  |

**Table S2 n.** The life table for females in 2018 using the model ‘JUV+2Y’ for estimation of qx.

| **x (years)** | **nx** | **lx** | **dx** | **qx** | **px** |
| --- | --- | --- | --- | --- | --- |
| 0 | **24** | 1.00 | 14.00 | 0.58 | 0.42 |
| 1 | **10** | 0.42 | 4.00 | **0.40** | 0.60 |
| 2 | **6** | 0.25 | 0.30 | **0.05** | 0.95 |
| 3 | ***6*** | 0.24 | 0.57 | **0.10** | 0.90 |
| 4 | ***5*** | 0.21 | 0.51 | **0.10** | 0.90 |
| 5 | ***5*** | 0.19 | 0.46 | **0.10** | 0.90 |
| 6 | ***4*** | 0.17 | 0.42 | **0.10** | 0.90 |
| 7 | ***4*** | 0.16 | 0.37 | **0.10** | 0.90 |
| 8 | ***3*** | 0.14 | 0.34 | **0.10** | 0.90 |
| 9 | ***3*** | 0.13 | 0.30 | **0.10** | 0.90 |
| 10 | ***3*** | 0.11 | 0.55 | **0.20** | 0.80 |
| 11 | ***2*** | 0.09 | 0.44 | **0.20** | 0.80 |
| 12 | ***2*** | 0.07 | 0.52 | **0.30** | 0.70 |
| 13 | ***1*** | 0.05 | 0.98 | **0.80** | 0.20 |
| 14 | ***0*** |  |  |  |  |

**Table S2 o.** The life table for males in 2017 using the model ‘JUV+2Y’ for estimation of qx.

| **x (years)** | **nx** | **lx** | **dx** | **qx** | **px** |
| --- | --- | --- | --- | --- | --- |
| 0 | **16** | 1.00 | 3.00 | 0.19 | 0.81 |
| 1 | **13** | 0.81 | 1.30 | **0.10** | 0.90 |
| 2 | **13** | 0.81 | 2.60 | **0.20** | 0.80 |
| 3 | ***10*** | 0.65 | 2.60 | **0.25** | 0.75 |
| 4 | ***8*** | 0.49 | 2.34 | **0.30** | 0.70 |
| 5 | ***5*** | 0.34 | 2.18 | **0.40** | 0.60 |
| 6 | ***3*** | 0.20 | 1.31 | **0.40** | 0.60 |
| 7 | ***2*** | 0.12 | 1.57 | **0.80** | 0.20 |
| 8 | ***0*** |  |  |  |  |
| 9 | ***0*** |  |  |  |  |
| 10 | ***0*** |  |  |  |  |
| 11 | ***0*** |  |  |  |  |
| 12 | ***0*** |  |  |  |  |
| 13 | ***0*** |  |  |  |  |
| 14 | ***0*** |  |  |  |  |

**Table S2 p.** The life table for females in 2017 using the model ‘JUV+2Y’ for estimation of qx.

| **x (years)** | **nx** | **lx** | **dx** | **qx** | **px** |
| --- | --- | --- | --- | --- | --- |
| 0 | **16** | 1.00 | 4.00 | 0.25 | 0.75 |
| 1 | **12** | 0.75 | 1.20 | **0.10** | 0.90 |
| 2 | **12** | 0.75 | 2.40 | **0.20** | 0.80 |
| 3 | ***10*** | 0.60 | 2.40 | **0.25** | 0.75 |
| 4 | ***7*** | 0.45 | 2.16 | **0.30** | 0.70 |
| 5 | ***5*** | 0.32 | 1.51 | **0.30** | 0.70 |
| 6 | ***4*** | 0.22 | 1.06 | **0.30** | 0.70 |
| 7 | ***2*** | 0.15 | 0.99 | **0.40** | 0.60 |
| 8 | ***1*** | 0.09 | 1.19 | **0.80** | 0.20 |
| 9 | ***0*** |  |  |  |  |
| 10 | ***0*** |  |  |  |  |
| 11 | ***0*** |  |  |  |  |
| 12 | ***0*** |  |  |  |  |
| 13 | ***0*** |  |  |  |  |
| 14 | ***0*** |  |  |  |  |
